# Validation of the "SmoCess-GP" instrument - a short patient questionnaire for assessing the smoking cessation activities of general practitioners: a cross-sectional study

**DOI:** 10.1186/1471-2296-11-9

**Published:** 2010-02-01

**Authors:** Julia Jung, Melanie Neumann, Markus Wirtz, Nicole Ernstmann, Andrea Staratschek-Jox, Jürgen Wolf, Holger Pfaff

**Affiliations:** 1 Institute for Medical Sociology, Health Services Research and Rehabilitation Science (IMVR), Faculty of Human Sciences and Medical Faculty, University of Cologne & Centre for Health Services Research Cologne (ZVFK), Eupener Strasse 129, Cologne 50933, Germany; 2 Medical Department of the University of Witten/Herdecke Integrated Curriculum for Anthroposophic Medicine (ICURAM); Alfred-Herrhausenstrasse 50, Witten 58448, Germany; 3 Institute for Psychology, University of Education Freiburg, Kunzenweg 21, Freiburg 79117, Germany; 4 Life and Medical Science Institute (LIMES), University of Bonn, Karlrobert-Kreiten Strasse 13, Bonn 53115, Germany; 5 First Department of Internal Medicine, Molecular Tumour Biology and Tumour Immunology, University Hospital Cologne, Kerpener Strasse 62, Cologne 50937, Germany; 6 Centre for Integrated Oncology (CIO), University Hospital Cologne, Kerpener Strasse 62, Cologne 50937, Germany

## Abstract

**Background:**

We developed an instrument assessing the extent of smoking cessation activities by general practitioners (GPs) within the Cologne Smoking Study (CoSmoS). The objective of the present study was to examine further psychometric quality of the "SmoCess-GP" instrument (**Smo**king **Cess**ation by **G**eneral **P**ractitioners).

**Methods:**

127 current smokers who had participated in the Cologne Smoking Study (CoSmoS) were included in our analyses. Confirmatory factor analysis (CFA) was conducted to examine the model fit and to retest the single-factor structure of the instrument using the Mplus software. Further construct validity was tested with bivariate analysis using an instrument which measures patients' trust in physicians.

**Results:**

CFA supported the unidimensional structure of the instrument. The factor loadings exceed the threshold of ≥ 0.50. All indicator reliabilities were higher than 0.30. The composite reliability was 0.86 and the average variance extracted (AVE) resulted in a value of 0.50. The calculation of global fit indices identified a CFI value of 1.00 and for TLI a value of 1.02. The root mean square error of approximation (RMSEA) indicates that 0% of the information is not accounted for by the model. The chi-square value was χ^2^_df = 6 _= 4.63 (p = 0.59). Analysis of discriminant validity resulted in a non-significiant correlation of r = 0.092 (p = 0.350).

**Conclusions:**

Results indicate preliminary evidence for the construct validity of the "SmoCess-GP" instrument which therefore appears to be a promising tool for analyzing the extent of smoking cessation advice offered by GPs from the patients' perspective. Future research should examine the psychometric properties in a population based sample, further improvements of the instrument and should apply other methods of validation.

## Background

In developed countries, smoking remains one of the most significant and preventable risk factors for cardiovascular and lung diseases. In Germany, in particular, approximately 37% of men and 28% of women smoke every day or on occasion [1]. Similarly, data collected within the European region as a whole demonstrate the same smoking prevalence [2]. Despite widespread knowledge of the risks and harmful consequences of smoking, this prevalence has remained nearly constant in Germany in recent years [3], whereas the smoking prevalence trends of other countries vary [4-7]. Nevertheless, over one billion people smoke worldwide [8], demonstrating the urgent need to increase and improve efforts in smoking prevention and cessation.

General practitioners (GPs) play a major role in the secondary prevention of smoking [9]. Acting as a so-called "gatekeeper," a GP has an intensive and, for the most part, long-term relationship with his patients. In large populations, it is a general practice that serves as a patient's first point of contact with the overall health care system. Therefore, this setting provides an effective means of accessing preventive care. Moreover, many smokers visit a GP on an annual basis, giving GPs the opportunity to promote smoking cessation interventions. For these reasons, general practice seems to be a promising setting for the implementation of professional preventive care.

According to German law, an important task of health care professionals - especially of GPs - is preventive care [10]. GPs should observe the law by providing patients with professional support. Such support includes attempts made to initiate, aid and sustain the efforts of their patients to be more health conscious [11], especially when it comes to quitting smoking.

Although special treatments for smoking cessation have increased in recent years [9] and the smoking prevention efforts of GPs have been proven effective [12-14], studies have shown that in various countries there is still only a small number of GPs who provide cessation advice to their patients during routine consultations [12,15,16]. The same holds true in Germany [17,18].

In Germany in particular, however, there is a lack of data concerning the extent and effectiveness of GPs' smoking cessation activities. According to Pfaff et al. [19], such data are necessary in order to describe and explain the professional care provided and in the next step to encourage and improve smoking cessation interventions. Following a systematic Medline search in March 2008, we identified only few validated instruments for collecting these data from the patients' perspective. For this reason, we designed a questionnaire within the context of the **Co**logne **Smo**king **S**tudy (CoSmoS). The present study evaluates the unidimensional structure of the "SmoCess-GP" (**Smo**king **Cess**ation by **G**eneral **P**ractitioners) instrument which could be found in a previous study [20] and to examine its further psychometric quality. The study makes first efforts toward confirming construct validity of the instrument with global and local fit indices using confirmatory factor analysis and it investigates its discriminant vailidity.

## Methods

### Measures

#### The "SmoCess-GP" measure

The "SmoCess-GP" instrument was carefully developed within the context of the CoSmoS study, a case-control study that examines which genetic and/or psychosocial factors lead to a higher risk for smokers to suffer from myocardial infarction, develop lung cancer and/or become addicted to nicotine.

In order to ensure content validity, internal consistency and the assessment of practice-relevant dimensions, the "SmoCess-GP" instrument was developed based on recognized international and German guidelines for the professional treatment of smoking dependence [13,21,22]. Brief interventions recommended for use by GPs in these guidelines were included as items in the instrument (see Table 1). "SmoCess-GP" retrospectively measures whether a particular intervention was undertaken by the treating GP. Based on cognitive pretests with a sample which consisted of n = 10 lung cancer patients, n = 10 myocardial infarction patients and n = 20 individuals from the general population of patients, the resulting "SmoCess-GP" instrument comprised six items (see Table 1) and had a dichotomous response format ("yes" = 1 or "no" = 0). Participants were also given the option of responding with "I do not consult a practitioner." Participants, who could not remember if their GPs had provided them with a smoking cessation intervention, could respond with "I don't know".

**Table 1:** Items of the "SmoCess-GP"- instrument and descriptive statistics (n = 127)

Item	Missing values	yes		no	
		n	%	n	%
1. My practitioner^1 ^frequently asked me how many cigarettes I smoke per day.	1	89	70.6	37	29.4
2. My practitioner frequently cautioned me about the negative consequences of smoking.	0	78	61.4	49	38.6
3. My practitioner empathically demanded me to quit smoking.	0	66	52	61	48
4. My practitioner recommended a smoking cessation course.	1	18	14.3	108	85.7
5. My practitioner gave me behavioral advice about quitting.	2	17	13.6	108	86.4
6. My practitioner prescribed me/advised me to undergo a nicotine replacement therapy (e.g., transdermal patches, nicotine solution drops, nicotine gum).	0	22	17.3	105	82.7

^1 ^The participants were asked to respond with regards to the practitioner they contact first if they have a health problem (e.g., their general practitioner).

An initial psychometric evaluation of the instrument was conducted using exploratory factor and reliability analysis as discussed in a previous study [20]. In summary, the findings in this study provided first indications of the measure being a reliable and valid instrument with an acceptable Cronbach's alpha of .68 [23].

#### Trust in physicians

With regard to construct validity, especially in order to examine convergent validity, within the context of CoSmoS it was not possible to integrate another, similar instrument for assessing smoking cessation activities by physicians. Therefore, as an example, the Health Plan Employer Data and Information Set (HEDIS) measure "Medical Assistance with Smoking Cessation" [24] would have been feasible. Thus, we approached the discriminant validity by analyzing the relationship of the "SmoCess-GP" instrument with the evaluation of the patients' trust in their GPs to ensure the measurement of different constructs. We used this variable because - in addition to "SmoCess-GP" - it also evaluates aspects of care by the patients' GPs. The construct was surveyed using the "trust in physicians_short form" scale of the Cologne-Patient-Questionnaire (CPQ) [25], which was developed and has been used in several research projects and studies carried out by the Institute for Medical Sociology, Health Services Research and Rehabilitation Science at the University of Cologne [e.g., [26,27]]. The scale was adapted for the ambulatory care [28] and it measures different aspects of a trusting physician-patient-interaction. The three items of the scale were worded as follows: (1) "I completely trusted my doctor", (2) "I had the impression that the doctor is very competent", and (3) "With the doctor one is in good hands". Four answer categories were given, ranging from "do not agree at all" (=1) to "completely agree" (=4). All items were summed and divided by the number of items.

#### Sample

The subjects of the present study were patients who had participated in the Cologne Smoking Study (CoSmoS) and who had been randomly recruited at the University Hospital Cologne.

The study was approved by the Ethics Committee of the University Hospital of Cologne (UHC). Patients were included in the study after signing an informed consent form. The participating patients suffered from either myocardial infarction or lung cancer or from neither of these conditions (control group). All patients smoked daily or on occasion up until the time of data collection. In face-to-face interviews, the participants were asked, in particular, about their smoking habits and the smoking cessation counseling provided by their GPs.

The sample was then reduced by excluding nine patients who stated that they had not consulted a general practitioner. This resulted in a total sample size of 127 currently smoking patients. The response "I don't know" was treated as a missing value.

The socio-demographic and illness-specific characteristics of our sample are presented in Table 2.

**Table 2:** Socio-demographic and illness-specific characteristics (n = 127)

Variable	n	Response trait	Frequency (n)	Percentage (%)
Disease	127	lung cancer	39	30.7
		myocardial infarction	41	32.3
		control group	47	37
				
Gender	121	male	78	64.5
		female	43	35.5
				
School education		with admission to higher education	30	23.6
		without admission to higher education	97	76.4
				
Professional education	121	with a graduate degree	102	84.3
		with a graduate degree	19	15.7
				
Age	117	*M = 54.8; Mdn = 55; SD = 9.5; Min = 35; Max = 75		
Age group	117	35-45	22	18.8
		46-55	42	35.9
		56-65	35	29.9
		66-75	18	15.4

*Note: *M = Mean; Mdn = Median; SD = Standard deviation; Min = Minimum; Max = Maximum

### Data analysis

#### Confirmatory factor analysis

The Statistical Package for Social Science (SPSS) Version 17.0 was used to compute the descriptive statistics and to obtain both the frequency and percentage distributions. The percentage of missing values for each item did not exceed 2.5%. In order to avoid biases, missing data were imputed using the NORM software, which uses the expectation-maximization (EM) algorithm to replace missing values [29].

Confirmatory factor analysis (CFA) was conducted to determine whether the instrument measures a unidimensional construct as intended. Due to the non-normal distribution resulting from dichotomous items, the analysis was conducted using tetrachoric correlations [30]. In addition, robust weighted least squares with the mean- and variance-adjusted χ^2 ^algorithm were applied using Mplus Version 4.2 [31].

CFA assumes that each manifest variable is a distinct indicator of an underlying latent construct. A CFA model is evaluated using measures of global and local fit. Such measures indicate whether the proposed model adequately reproduces the empirical correlations among the manifest variables [23].

For several of the recommended global fit measures, certain criteria must be met in order to accept the model as being plausible and parsimonious. Measures of absolute fit, such as the root mean square error of approximation (RMSEA), can be interpreted as the amount of information within the empirical covariance matrix that cannot be explained by the proposed model. The model may be classified as acceptable if only 8% or less of the information is not accounted for by the model (RMSEA < 0.08). Other goodness-of-fit indicators, including the comparative fit index (CFI) and the Tucker-Lewis index (TLI), were also examined. Hair et al. [23] suggest using adjusted index cut-off values based on model characteristics. They maintain that simple models and small samples should be subject to stricter evaluation than more complex models with larger samples. Based on their recommendations, we used a sample size of N < 250, a number of observed variables ≤ 12, a CFI and a TLI of 0.97 or better, and a RMSEA < 0.08.

The process of psychometric evaluation also involves testing the reliability of measurement and assessing the quality of the individual items, both of which are evaluated using component fit results (e.g., factor loadings, composite reliability, average variance extracted and indicator reliability) from the confirmatory factor analysis. Measures of local fit evaluate whether each construct can be reliably estimated from its indicators [23]. The factor loadings - recommended to be at a value of 0.5 or higher - indicate how much of the variance in an item is explained by the latent factor. In addition, it is crucial to examine the reliability of the proposed model, especially its composite reliability, for which 0.6 is an acceptable value. The average variance extracted (AVE) is a summary measure of convergence among the items. Its recommended acceptable threshold is ≥ 0.5. Indicator reliability should be greater than 0.3 in order to guarantee a stable estimate [23].

#### Discriminant validity

In addition to CFA, we tried to examine construct validity with further analysis. In the context of the present study it was possible to test the discriminant validity of the "SmoCess-GP" instrument as one form of construct validation [32]. Therefore, the relationship with the patients' trust in their GPs was analyzed. We used bivariate analysis (Pearson's correlation) with the mean sum score of the "SmoCess-GP" instrument and the mean sum score of the "trust in physicians_short form" scale. A weak association was expected between both constructs because of assumed distal relationships between those constructs.

The SPSS Version 17.0 for Windows software was used to conduct the analysis for construct validity.

## Results

### Descriptive findings

The descriptive statistics for each item of the "SmoCess-GP" instrument is shown in Table 1. As a whole, not all participants had been screened by their physicians to determine the extent of their smoking habits. In fact, there is a noticeable decrease in the percentage of smokers in all disease groups, who received physician-delivered interventions as of item 4. This means that only a small proportion of smokers were offered special smoking cessation interventions, such as being recommended a smoking cessation course (item 4), being provided with advice on quitting (item 5) or being prescribed a nicotine replacement therapy (item 6). The mean of approximately 2.3 (range: 0-6: 0 = no intervention at all, 6 = all 6 interventions; median = 2; standard deviation = 1.58) indicates that the number of smoking cessation interventions offered by GPs is, as a whole, small and that patients were unable to obtain concrete assistance and support.

In addition, the descriptive findings of the "trust in physicians_short form" scale, which is used for examining the discriminant validity of the "SmoCess-GP" measure, are presented in Table 3.

**Table 3:** Descriptive statistics of the "trust in physicians_short form" (TRIP_sf) scale

Item	Missing value	M	SD	Min	Max
1. I completely trusted my doctor	6	3.52	0.856	1	4
2. I had the impression that the doctor is very competent	6	3.50	0.845	1	4
3. With the doctor one is in good hands	6	3.50	0.845	1	4
TRIP_sf	6	3.50	0.793	1	4

*Note: *The participants were asked to respond with regards to the practitioner they contact first if they have a health problem (e.g., their general practitioner).

Answer categories ranging from "do not agree at all" (=1) to "completely agree" (=4).

M = Mean; SD = Standard deviation; Min = Minimum; Max = Maximum

### Confirmatory factor analysis

CFA of the "SmoCess-GP" measure found that the same unidimensional structure of the six items was replicated as in the exploratory factor analysis in our pilot study [20]. To quantify the differences between observed and estimated covariance matrices [23], the chi-square value was calculated. With χ^2^_df = 6 _= 4.63 and a nonsignificant p-value of 0.59, a very good model fit was obtained [23]. The model, therefore, adequately accounts for the information in the empirical covariance matrix. In addition, the global fit indices, such as the CFI with a value of 1.00 and the TLI with a value of 1.02, show very good values. The root mean square error of approximation (RMSEA) of 0.00 indicates that 0% of the information is not accounted for by the model, which also demonstrates the excellent fit of our model. The results of the analysis can be found in Table 4.

**Table 4:** Measures of global fit

	χ^2^_df_	df	p	TLI	CFI	RMSEA
*Thresholds for acceptable fit*			*>.05*	*>0.97*	*>0.97*	*<0.08*
CFA model	4.63	6	0.59	1.02	1.00	0.00

TLI = Tucker-Lewis index; CFI = comparative fit index; RMSEA = root mean square error of approximation. For more on acceptable fit, see [23].

Examination of the factor loadings reveals that all six items have significant loadings on the construct in the model and exceed the threshold of ≥ 0.50 as recommended (see Table 5). All six indicator reliabilities with values higher than 0.30 (see Table 5) exceed the acceptable values [23]. The same is true for the composite reliability (0.86) and the average variance extracted (AVE = 0.50).

**Table 5:** Factor loadings and indicator reliabilities

Item	Factor loadings	Indicator reliabilities
*Thresholds for acceptable values*	*(>0.5)*	*(>0.3)*
1*	0.71	0.50
2	0.78	0.62
3	0.78	0.61
4	0.77	0.59
5	0.58	0.33
6	0.63	0.40

*The wording of the items is shown in Table 1

### Discriminant validity

In terms of discriminant validity of the "SmoCess-GP" instrument, the bivariate analysis with the "trust in physicians_short form" scale resulted in a correlation of r = 0.092; p = 0.350.

## Discussion

We developed a short instrument within the context of the Cologne Smoking Study (CoSmoS) for assessing the extent of smoking cessation interventions provided by GPs as seen from the patients' perspective. The present study evaluated the psychometric quality of the "SmoCess-GP" measure using confirmatory factor analysis (CFA) in order to analyze its unidimensional structure. The analysis indicated a very good model fit. Both the resulting chi-square value with a non-significant p-value as well as the CFI, TLI and RMSEA values provide evidence of an adequate global data fit. The local fit indices support the explicitness of the "SmoCess-GP" instrument, and the indicator reliabilities prove that the items reliably measure the construct. Acceptable factor loading values, composite reliability and the average variance extracted confirm the quality and reliability of the measure and its items. As confirmed by all of the goodness-of-fit indices, it was possible to replicate the unidimensionality of the instrument.

In terms of discriminant validity, the results of the bivariate analysis confirmed the initial hypothesis that there is only a weak relationship between the patients' trust in their GPs and the provision of smoking cessation counseling by them.

Our findings can therefore be seen as preliminary indicators of the construct validity of the "SmoCess-GP" instrument which has to be further evaluated using other forms and methods of validation.

The descriptive findings - in specific, the mean sum score - of the "SmoCess-GP" instrument indicate a lack of preventive care in German general practices because not every smoking patient in our sample received anti-smoking advice from their GPs and because several different concrete intervention options were seldom offered. However, these results are not unique to Germany. Other international studies have reported similar findings [12,15-18], and the lack of preventive care has often been explained by structural barriers, such as reimbursement [e.g., [33]], the medical setting or training [e.g., [34]].

### Limitations of the study

Several limitations of the present study have to be mentioned. First of all, given the retrospective design of the study and the hospital setting, there may be a bias caused by distortions in the patients' memory. Although participants were asked to think of their general practitioner when responding to statements, it is not known exactly which physician and which consultation experiences the patients were actually remembering when being surveyed. Furthermore, in view of the findings of Houston et al. [35], which indicated that delayed measures of provider performance from the patients' perspective, like the HEDIS, may result in an over- or under-estimation, we can not exclude that the "SmoCess-GP" instrument also suffers from this limitation. In this regard, a possible over-reporting of smoking cessation interventions by GPs in our study can also be caused by the wording of the "SmoCess-GP" instrument, which asks for provided counseling in general, without a specific time-frame of consultations. A more time-specific query will certainly lead to different patient responses and should be tested in comparative studies.

Second, the face-to-face interview method may also have been a source of bias. An interviewer and other patients in the hospital room may have prompted participants to provide false or socially desirable responses. Third, the convergent validity of the "SmoCess-GP" instrument could not be tested against similar measures because a comparable instrument had not been integrated in the CoSmoS questionnaire.

The generalizability of our findings is limited because the sample was inpatient and thus no conclusions for the population can be drawn.

### Future research

Future research should evaluate the instrument psychometrically in prospective studies with a population-based sample. In addition, the convergent validity should be tested with similar smoking cessation intervention measures like for example the Health Plan Employer Data and Information Set (HEDIS) measure "Medical Assistance with Smoking Cessation" [24]. Also concurrent validity can be evaluated with observation studies or studies in which consultations are audio taped. Audio taping is regarded as a more objectively assessment of the physicians behavior than patients surveys [36,37]. Thus, the results of our study must be interpreted with caution, because an over- or under-reporting can not be excluded so far. On the other hand there are several other studies which have examined the concurrent validity of physicians' behavior and patients' recall. For example, the results of Ward and Sanson-Fisher [38] indicated a high sensitivity (92%) and a somewhat lower specificity (82%) of patients' recall of inter alia smoking cessation advices by a post-graduated trainee. Stange et al. [37] evaluated the validity of non-observational methods, such as patient questionnaires for assessing inter alia smoking cessation counseling services with observational methods. Findings showed an acceptable accuracy (sensitivity of 72% and specificity of 96%) of the patients exit surveys. The observations of smoking counseling activities by physicians in the survey of Ellerbeck et al. [39] showed good correlations with the patients' recall on this topic. Wilson and Mc Donald [36] found an acceptable sensitivity (81.8%) in estimating smoking cessation counseling for smoking patients by patient questionnaires compared with the detection on audio tape. The rate of false positive answers was 10.5%. The authors conclude, that "a patient questionnaire may be the most feasible method of assessing in large scale research or audit studies whether advice on lifestyle...has been given." [[36], p.1485]. Moreover, a valid correlation with the assessment of smoking cessation interventions from the GPs' perspective would provide additional evidence for criterion validity.

Following the practice guideline of the U.S. Department of Health and Human Service, smoking cessation interventions should be adapted to the smoker's stage of readiness [40]. Therefore, the third item "My practitioner empathically demanded me to quit smoking" should be modified for further studies towards a more motivational than demanding meaning. This should be carried out on the basis of the recommendation of the guideline which points out, that in case of patients unwilling to quit Motivational Interviewing (MI) "...is more effective than clinician exhortations, lectures, or arguments for quitting..."[[40], p.57].

In the present study, we did not ask about how often the smoking cessation counseling was provided. This could be of interest in this context and should be considered in further development of the instrument.

## Conclusion

Our aim was to develop a measure, which describes and evaluates the smoking cessation interventions in general practice. Despite the study's limitations and requirements of valuable improvements of the instrument, the findings indicate reasonable results in terms of the preliminary validity and reliability of the new "SmoCess-GP" instrument. As a patient-reported measure, it provides researchers and GPs an instrument that makes it possible to analyze the extent of smoking cessation interventions in general practices.

### Practice implications

Although the GP plays a major role in preventive care, the change of unhealthy behavior is often considered to be a matter of personal choice [11]. Despite evidence for the effectiveness of smoking cessation interventions provided by GPs [12-14], their full potential has not yet been exploited [12,15-18]. For this reason, more support should be given to the preventive activities of GPs.

The theoretical and empirical basis of the "SmoCess-GP" measure (see also [20]) provides a useful tool for research as well as a timesaving checklist for guiding GPs through smoking cessation counseling. As such, the instrument can be used for GP self-audit and to help to identify gaps in interventions. The "SmoCess-GP" instrument also seems appropriate for use by other healthcare professionals (e.g., specialized physicians or nurses).

## Abbreviations

GP: general practitioner; SmoCess: smoking cessation; CoSmoS: Cologne Smoking Study; CFA: confirmatory factor analysis; RMSEA: root mean square error of approximation; CFI: comparative fit index; TLI: Tucker-Lewis index; AVE: average variance extracted; M: mean; Mdn: median; SD: standard deviation; Min: minimum; Max: maximum; OR: odds ratio.

## Competing interests

The authors indicated no potential conflict of interest.

We confirm all patient/personal identifiers have been removed or disguised so that the patient/person(s) described are not identifiable and cannot be identified through the details of the story.

## Authors' contributions

JJ conducted data collection and analysis, and wrote the first draft of the manuscript. MN contributed to study design, editorial review and data analysis. NE contributed to study design and editorial review. MW contributed to data analysis, technical, including statistical and editorial review. ASJ and JW contributed to study design and editorial review. HP contributed to study design and editorial review. All authors read and approved the final manuscript.

The corresponding authors take full responsibility for the content of the paper.

## Pre-publication history

The pre-publication history for this paper can be accessed here:

http://www.biomedcentral.com/1471-2296/11/9/prepub
